# Long-term impact of Global Health educational experiences in Rome: an attempt of measurement

**DOI:** 10.1186/s13690-020-00478-z

**Published:** 2020-10-02

**Authors:** Giulia Civitelli, Gianfranco Tarsitani, Alessandro Rinaldi, Maurizio Marceca

**Affiliations:** Department of Public Health and Infectious Diseases - Sapienza University of Rome, Piazzale Aldo Moro 5, 00185 Rome, Italy

**Keywords:** Global health education, Health equity, Migrants, Refugees, Prisoners, Third-Mission

## Abstract

**Background:**

Global health education (GHE) is spreading in Europe and in other parts of the world. Since 2008, Sapienza University of Rome has offered activities to medical and other health profession students related to global health (GH), which is grounded in the theory of social determinants of health and inspired by social justice. The educational activities included elective courses as well as community and service-learning experiences, referred to as GH gyms. This study attempts to measure the long-term impact of these educational experiences, especially to demonstrate their influence on the perceived social responsibility of future health professionals.

**Methods:**

A questionnaire was elaborated and tested on a small sample of participants. It was sent to participants by e-mail. Quantitative results were analysed through descriptive statistics and qualitative answers were carefully read and classified.

**Results:**

A total of 758 students from different faculties took part to the educational experiences. Only 488 e-mail addresses were available. One hundred and five (21.5%) questionnaires were returned. Participation in GH gyms was perceived to have had a higher influence on future professional and personal choices, when compared to participation in elective GH courses.

**Conclusions:**

The study shows that consideration of health and social issues related with inequities in health and the use of interactive teaching methodologies had important effects on social responsibility of a large number of students. As there could be a selection bias among respondents, more research is needed to understand the impact of GH educational experiences. The inclusion of global health education in health and social curricula and the use of interactive methodologies with a correct evaluation of results are the indications that emerge from this research, together with the necessity of a strong involvement of students, professors and the whole academic reality.

## Background

Global health education (GHE) is becoming more important for the education of current and future health professionals, as a means to prepare them to live and work in a globalized world [1–19]. The Global Health approach, based on the theory of social determinants of health, aims to allow physicians to recognise and tackle the social, economic, political and cultural factors which influence patient’s health. Those factors, related to the local, national and global context, are the causes of existing inequities in health within and between countries. The authors argue for the importance of including social medicine and global health in the preclinical curriculum [20] (or in undergraduate degree for US medical schools) and underline that more work needs to be done to explain the relevance of global health (GH) to medical students from the outset of their studies [21].

Especially with the rise of reactionary nationalism putting a strain on social cohesion, [22] it is important to act in the political and academic processes to promote the concrete implementation of GH aspects within and beyond the medical curricula [23]. GHE has the potential to be an example of transformative education, which is the proposed outcome of instructional reforms presented by the Lancet Commission’s *Education of Health Professionals for the twenty-first Century* [24].

In Italy GHE began to spread in 2007 thanks to the European project “*Equal opportunities for health*”, coordinated by the NGO ‘Doctors with Africa CUAMM’. Before this project few universities offered courses in this field. The project led to the development of the Italian Network for Global Health Education (INGHE, RIISG in the Italian language), a network of Universities, Scientific Societies, Non-Governmental Organization and Medical Students’ Associations interested in the promotion of GHE at undergraduate and postgraduate level [25–28].

At Sapienza University of Rome, the first GH elective course was organized in January 2008 at the Department of Public Health and Infectious Diseases (formerly, Department of Public Health Sciences). A GH elective has been organized every year since, while the characteristics and organization of the course has been refined and detailed. Since 2009 a second edition of the GH course has been organized every year at St. Andrew Hospital, headquarters of the Second Faculty of Medicine and Surgery at Sapienza University of Rome.

The main topics of the course were those recognised by INGHE as the fundamentals of GH: social determinants of health, inequities in health, globalization and health, health systems, migration and health and international health cooperation. Every year the course was offered not only to medical students, but also to students of other health and social professions. Interdisciplinarity is a characteristic of GH and sharing the same classroom and lessons is a first step in learning how to collaborate in the future, with a view to work towards better health for all. The course is elective and may be counted towards university credit, according to the degree course syllabus.

A university professor of public health was initially the main organiser of the course, structured as a series of lectures given by experts. Over the years a group of students and residents, who were particularly interested in GH, became the main promoters of the course, with professorial supervision. Gradually, thanks to the involvement of students and to the interest of the academic staff, peer education methods have been introduced and enhanced. The young scientific committee introduced the direct recounting of important experiences, group activities, role-play and other interactive educational methods.

The academic staff utilised an evaluation system from the beginning of the course: a knowledge pre-test, the collection of the expectations of participants, a satisfaction evaluation form after each day of the course and an overall satisfaction evaluation form at the end of the course Learning evaluation was realized through post-tests and, in some cases, with the request for participants to write a short paper on one of the topics.

The Department of Public Health and Infectious Diseases also organised community education experiences [29, 30], in collaboration with the Secretariat of Italian Medical Students (SISM) and associations that work in the context of exclusion and marginalization. In Italy, these types of community experiences are referred to as “GH gyms”, as they allow students to experience different realities, test and train themselves in contexts different from the academic one. In GH gyms, students have the possibility to:

- observe the circumstances of asylum seekers and refugees, thanks to a project organized with the Italian Jesuit Refugee Service Italy (a Catholic organisation with a mission to accompany, serve, and advocate on behalf of refugees and other forcibly displaced persons);
- engage with undocumented migrants’ health services, in collaboration with Caritas Medical Area, Rome (the health sector of Caritas Rome, which is the Pastoral Body of the Church of Rome in order to promote the charity commitment of the Italian community);
- explore the reality of prisoners’ health, in collaboration with Antigone, a cultural and political NGO.

The organizations mentioned above has been chosen as partners because with the academic staff there was a reciprocal knowledge and a consolidated collaboration. The organizations were also used to train and involve young volunteers in their services.

Students who want to take part to a GH gym are suggested to attend before a GH course, but it is not mandatory. The three types of GH gyms are detailed below.

There are very few literature sources regarding educational experiences related to migrant and refugee health [31–35]. The GH gym project ‘Know the reality of asylum seekers and refugees’ offered medical students and students of other health and social professions the unique possibility to enter into the physical space of a migrants’ shelter. The experience was introduced by a nine-hours course (three meetings, each of 3 h): the first meeting took place at the university and introduces the GH approach to migrant health issues; the second took place at the headquarters of the Jesuit Refugee Service in Rome and introduced the reality of asylum seekers and refuges, as well as the work of the Catholic supporting organization; the final meeting took place at a migrant shelter and included an explanation of the contexts in which students could be present and enter in relationship with migrants. Students were then asked to be present in the shelter one afternoon a week and to be available for those migrants who wanted to improve their Italian language skills. This was not a specific health task, but for students it allowed an occasion to understand the context of life and the stories of migrants and to see how several factors influence their health. Students were invited to write a diary of the experience and, only on a voluntary basis, to share their observations with other participants. In addition, periodic meetings were organized with the academic supervisors, in order to reflect about social determinants of health and consider how the experience might change the ethical vision of future health professionals. Thirty-four students took part in a total of five editions of the project.

Another project, “*On the tracks of the right to health”,* was realized in collaboration with the health department of the Catholic organization Caritas of Rome in 2012/2013. Ten medical students took part in the experience, which was conceived as a “second level experience” after the participation in an introductory course to GH and migrants’ health. Students were asked to map all the undocumented migrants’ health services of the National Health System in Rome. A questionnaire was devised to collect information such as opening times, accessibility, documents requested, languages spoken by health and administrative professionals, and the presence of cultural mediators. Students were invited to reach these services using the public transport services that many migrants rely on. Students were also invited to write a diary of the experience, describing both the services and their involvement in the project. The results of the project consisted of an up-to-date map of undocumented migrants’ health services in Rome, available to all interested parties, and a publication with the analysis of the student diaries.

Finally, the project “*Health in prison*” was realized in collaboration with Antigone, a cultural and political NGO. It allowed medical students the chance to experience the reality of prison conditions. This experience recalled the work of Brooker and colleagues [36], who showed how an internship in a prison health system could stimulate career interest in an under-served area. In Italy, lawyers and physicians can operate voluntarily in some Italian prisons, giving consultations to prisoners and monitoring life conditions. In two editions of the “Health in prison” project, 13 students from different backgrounds took part to the experience where, following an introductory course, they were able to follow the already embedded voluntary team of the association in the prison activities. Students were engaged in collecting and evaluating requests for assessments of prisoners’ health care needs. Periodic meetings were organized with the academic staff to share and supervise the experience.

The main objectives of GH courses and GH gym were to educate future social and health professionals to become aware of their social responsibility and gain insights needed to tackle health inequities and promote social justice. Nevertheless, there are few studies that investigate the long-term impact of educational experiences [29, 37]. This study tries to add to an understanding of the long-term impact of those kind of educational experiences on the personal and professional choices of students at the early stages of their career.

## Methods

A literature non-systematic review has been conducted on PubMed, Scopus and Web of Science using two groups of terms, connected with Boolean operators: a first group of terms related with GH/ Inequities in health/ Social Determinants of Health/ Migrants health and a second group of terms related with academic education. Criteria of inclusion were articles which describe undergraduate educational experiences related with GH topics and realities. A questionnaire was developed with closed-ended and open-ended questions using the Likert scale evaluation. The questionnaire was tested by submitting it to a small panel of students who at the beginning took part to GH courses and gym as participants and then they become promoter and organizer of the educational experiences. The test aimed to evaluate if the questionnaire was comprehensible, sustainable and complete.

The authorization to perform the research was granted by the Department’s Council and the Ethics Committee of Sapienza University of Rome was informed. The questionnaire was sent by e-mail to the participants, together with informed consent and data treatment authorization agreements. Email addresses were taken from the list of enrolment of each GH course and gym. Respondents could stay anonymous. Answers were collected over a period of time of 4 months.

The questionnaire investigated the characteristics of the responder (sex, year of birth, nationality, degree course, current situation of study, work or other) and their participation to GH courses or GH gyms. Both those educational experiences are elective and participation is student’s choice. For GH courses, the most appreciated topics and didactic methods were investigated. For GH gyms, the questionnaire allowed for narrative commentary if there were aspects that were particularly important for the participant. Final questions for both courses and gyms used the 6-point Likert scale with questions of agreement to explore the influence of educational experiences on personal and professional choices. The last question (of agreement) used a 10-point Likert scale to explore the influence of those experiences in promoting personal responsibility towards social justice and in tackling health inequities.

Collected data was analysed trough excel (Microsoft Office version 16.0.11629.20246), using descriptive statistics. Variables have been expressed in absolute and percentage frequencies. A comparison analysis between participants to GH courses and participants to GH gyms has been made to look for differences in influence of these two types of educational experiences.

## Results

Since the beginning of the initiative, 758 students from more than 10 faculties participated in GH courses (Table 1). The high majority of participants (82.4%) were female.

**Table 1:** Participants to Global Health academic courses from 2008 to 2018

Degree course	Total	Male	Female
Social service	259	14	245
Medicine	230	69	161
Rehabilitation Sciences	110	23	87
Nursing	101	25	76
Obstetrics	37	1	36
Psychology	13	1	12
Others	8	0	8
Total	758	133 (17.5%)	625 (82.4%)

Of the total of 758 students who took part to the GH courses, it was possible to recover and use a total of 433 e-mail addresses (some email addresses were not available and others were no more functional, as the questionnaire was sent back to the sender).

55 students took part in GH gyms, two of whom took part in two different gyms. It was possible to recover all the e-mail addresses of GH gym participants (Table 2).

**Table 2:** Participants to Global Health gyms from 2012 to 2017

Project	Total
“*Know the reality of asylum seekers and refugees*”	34
“*On the tracks of the right to health”*	10
“*Health in prison*”	13
Total	57^a^

^a^2 students took part to two different GH gyms

The authors received 105 answers in total (21.5% of the total 488 e-mails sent) and a descriptive analysis of the sample is described in Table 3.

**Table 3:** Demographic characteristics of survey participants

**SEX**		
Male	26	24.8%
Female	79	75.2%
**AGE**		
21–25	18	17.1%
26–30	45	42.9%
31–35	22	21.0%
36–40	8	7.6%
41–45	4	3.8%
46–50	6	5.7%
51+	2	1.9%
**DEGREE COURSES**		
Medicine	43	40.9%
Social Service	19	18.1%
Rehabilitation Sciences	17	16.2%
Nursing	15	14.3%
Obstetrics	4	3.8%
Psychology	3	2.9%
Others	4	3.8%

As discussed later, there could be a selection bias: the authors are aware that respondents could be the most interested and involved students. This should not be considered a good reason to ignore and not analyse the results. The main arguments of this article are medical education, students and their future commitment to tackle health’s inequities. It is known that, in the field of education, changing knowledge, skills and attitudes even of a small group of people could result in important changes for the society. For this, the authors believe that it is worthy to reflect on the answers received and present the results to the international community.

For what concerns a possible sex bias, it could be explained on one hand with the higher number of female students in Medical Schools (around 58% according to the Italian Ministry of Education) and on the other hand with the higher interest and sensitivity girls have for GH topics.

Among the 105 participants to the survey, 79 took part only in GH courses (18.2% of the participants to GH courses reached through e-mail), and 26 in GH gyms (47.3% of the participants to GH gyms reached through e-mail). Of the 26 participants to GH gyms, 25 took part also to GH courses. In Table 4 is described the year in which research participants performed the GH educational experience.

**Table 4:** Year in which research participants performed the Global Health educational experience

	GH courses		GH gym	
2007	2	1,9%	/	/
2008	2	1,9%	/	/
2009	1	1,0%	/	/
2010	3	2,9%	/	/
2011	2	1,9%	/	/
2012	3	2,9%	/	/
2013	3	2,9%	/	/
2014	9	8,7%	6	23,1%
2015	12	11,5%	6	23,1%
2016	15	14,4%	8	30,8%
2017	17	16,3%	2	7,7%
2018	30	28,8%	2	7,7%
MD	5	4,8%	2	7,7%
Total	104	100,0%	26	100,0%

**Fig. 1:**
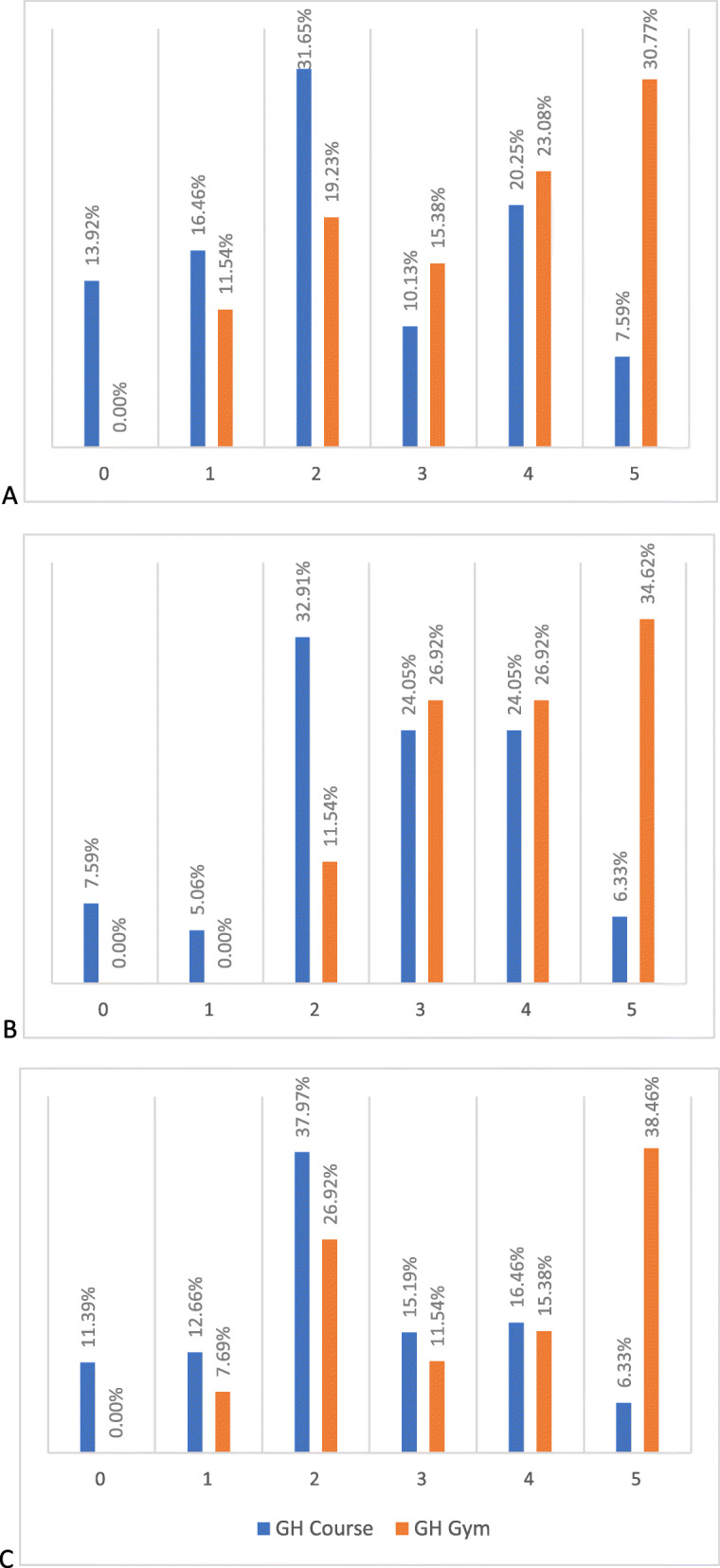
Influence of GH educational experiences on study, personal and professional choices. Answers to the questions *Do you believe that these types of educational experiences have influenced your study choices/ personal choices/ professional choices? Select a value from 0 to 5, where 0 is not at all, 1 is very little, 2 is sufficient, 3 is a lot, 4 is very much, and 5 is absolutely*. (**a**: “Influence on study choices” **b**: “Influence on personal choices” **c**: “Influence on professional choices”) Legend: GH course vs GH gym

**Fig. 2:**
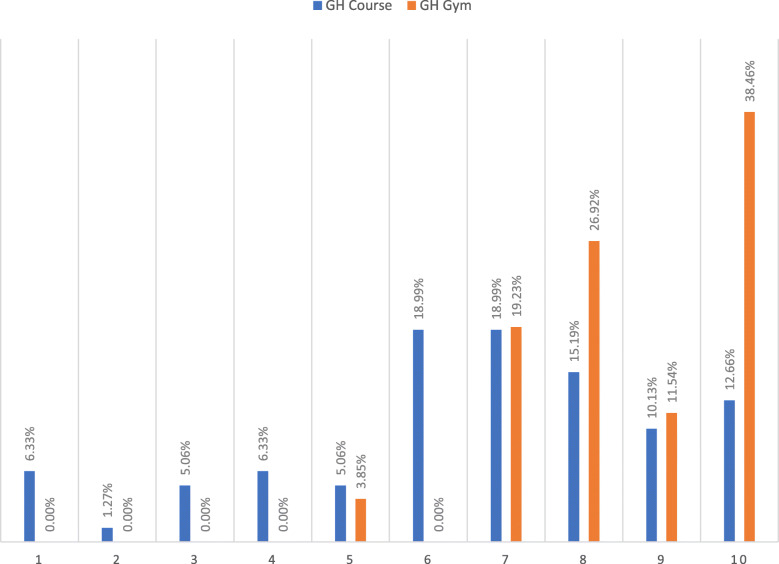
Overall influence on social responsibility. Answers to question: *Overall, how much have you been influenced from those educational experiences in taking a position for social justice and in acting against existing health inequities? Select a value from 1 to 10, where 1 is “no influence” and 10 is “biggest/ highest influence”.* Legend: GH course vs GH gym

Analysis of the results of the questions that investigate the long-term impact of the experience clearly showed that students who took part in a GH gym were more influenced by this experience for their future life (Table 5). The percentage of GH gym participants who selected a high value to express the influence of the experiences on their academic, personal and professional choices (Fig. 1) and on their overall social responsibility (Fig. 2) is greater than the percentage for GH course participants.

**Table 5:** Mode of the influence on different types of choices of Global Health Educational experiences

	GH courses	GH gyms
Academic choices ^a^	2	5
Personal choices ^a^	2	5
Professional choices^a^	2	5
Influence on overall social responsibility ^b^	6–7	10

^a^6-point Likert scale from 0 to 5

^b^10-point Likert scale from 1 to 10

As concerns GH courses, it is interesting to note that the most appreciated teaching method was witnesses/testimonies, which was mentioned by 73/104 people. The most appreciated topics were social determinants of health, migration and health, health in prison, housing as social determinants of health, the experience of Bastogi, a suburb of Rome.

GH gyms students reported being most impressed by the opportunity to enter in contact with a “*reality that I felt far away from me*”, the opportunity to “*go out from classrooms and learn in real life a different concept of health*”, the opportunity to enter in relationship with migrants and prisoners and to discover their underlying humanity. The importance of group work in orienting education towards a global vision was also mentioned.

Excerpts of some particularly significant experiences are detailed below.

*Dialogues with asylum seekers and refugees allowed me to wide my knowledges and to know their stories and their future projects.*

*It was a unique experience. It is difficult to explain it in few words.*

*I was impressed mostly by the meeting with migrants and the interaction between different social and health professionals.*

*It is difficult to describe the experience in prison in few words. What impressed me the most was to see how in some places, characteristics or roles of a person became more important than the fact that he or she is a human being.*

*I was mostly impressed by the interaction with refugees, their life-stories and the tale of their travel/ migration process.*

*I was impressed by the strength of the initiative, the group work which oriented my education towards a global vision.*

*I was impressed by the direct and personal relationship with people, as refugees or prisoners, beyond the theorical and scientific aspect of the course. The relationship was a strong incentive and a source of knowledge.*

*I was impressed by the possibility to go out from classrooms and learn in the real life a different concept of health.*

*I was impressed by the possibility to know a reality that I felt far away from me.*

*I was impressed by the possibility to know the reality of the prison and to work in this reality*.

*I was impressed by the situation of big social and psychological fragility of refugees, people who were already tested by a difficult past life and who often don’t see a future in front of them. This experience allowed me to enter in contact with strangers, people otherwise extraneous from my life, and often considered only as a threat. The most important thing that I received was a sense of proximity with those people, who before I perceived as distant from me. Curiously, what impressed me the most was to discover that their dialogues are not different from ours…the same dreams, fears, fragilities…It was very important for me. Before this experience, hearing Arabic in the street gave me a sense of mistrust. Now I know that behind those incomprehensible words maybe there is somebody who is discussing about what to prepare for dinner at home.*

Some final overall impressions and comments from students are reported below.

*Maybe it is thanks to this kind of initiatives that, even with strain/difficulty, I managed to arrive at the end of the academic course, which was drying me up.*

*I would like to thank you because I needed all those things and I’ve been lucky to meet you.*

*Those educational experiences enriched my already existing basis of awarness and political activism.*

*It was a very interesting course. I will always have a beautiful memory of it.*

*Go forward with enthusiasm and perseverance in those kinds of initiatives.*

*We should struggle together for the safeguarding of the common goods.*

## Discussions

It has been shown that medical schools can play active roles in alleviating the physician shortage in underserved areas through targeted curricular interventions and recruitment [38]. The aim of the study was to investigate the long-term impact of educational experiences in GH made by students of different degree courses at Sapienza University of Rome. This is the first Italian survey of the subject.

The most important finding is related with the higher impact of participation in GH gym on personal and professional choices compared with the participation in GH courses. This is underlined first, by the answer rate among those who took part to courses and gyms, and secondly, by the students’ high attribution of the influence of gyms on the growth of personal social responsibility compared to those who only took part in GH courses. Entering into contact with reality, entering in relationships with marginalized people and with services and professionals committed to defend the right to health – which means the right to well-being – are important ways to increase the awareness of personal and social responsibility.

The findings indicate that educational experiences outside classrooms, in places where it is possible to enter in contact with marginalized people, are effective ways to stimulate students to take positions in support of social justice and against health inequities. GH gyms may be not only a good example of transformative learning, whose aim is to produce change agents “with the status, authority, and ability to promote enlightened transformation in society” [24], but also a concrete project of the University’s Third Mission. An interesting characteristic of these educational experiences is the collaboration with non-governmental organizations, which work directly in the field. This kind of partnership “can create a feasible, effective, and sustainable platform for teaching medical students about the social determinants of health” [39]. If the aim of the university is to educate future professionals who are aware of the society in which they should live and work, going out from the university and spending time with underserved people, in a supervised academic project could be a good way to reach this aim.

The authors were also impressed by the selection of witnesses/ testimonies as the preferred didactic method. This result shows clearly that what remains as the medium- to long-term impact of educational courses are the moments in which a knowledge is related with emotion.

### Limitations

of the study are related with the answer rate compared with the total population who took part in GH educational experiences. This could be related to e-mail addresses that were not in use after some years. Considering that the first GH course took place in 2008, it was not possible to find all the current e-mail addresses of every participant. The survey submission deadline was a second probable reason for the low answer rate. Other limitations of the study could be related to a selection bias: we could suppose that participants who answered the survey were those most motivated even before taking part to the educational experiences. This should not be considered only as a limitation. Even if it is true that among students there are those more interested and committed for social justice for personal reasons, this is not a good argument to consider GH educational experiences as disadvantageous or unimportant. On the contrary, some former students, who are now health and social professionals, made life and professionals choices which are coherent with the vision of GH and commit themselves to tackling health inequities at national or international level.

More research is needed to better understand the concrete impact of these educational experiences and possible ways to promote them. Two main obstacles may be considered. The first is related to the possibility of inserting similar educational proposals in the wider academic curriculum. These experiences grew from the personal interest of an academic professor and the involvement of students and residents, and it would have to be shown whether a similar program with different actors could have similar outcomes.

The second potential obstacle to broader distribution of the initiatives relates to the participation of students. Initiatives would need to be made to invite even those who are apparently not interested in the GH approach and may be reluctant to enter into contact with different realities, especially realities of exclusions and marginalization.

## Conclusions

The continuous development of technology applied to medicine is inevitably deflecting the attention from a wide view of education for health to the acquisition of more and more complex technical knowledge and skills. In this context it could be interesting to identify teaching methodologies that are able to influence students of social and health professions in their future professional and personal choices. The results of this study go in this direction: even if the numbers are limited, they show the long-term impact of GH courses and, even more, of GH gyms on student attitudes. Analysis of health and social issues related with inequities in health and the use of interactive teaching methodologies had important effects on a large number of students. This is clearly demonstrated by the written comments of students. The inclusion of GHE in health and social curricula and the use of interactive methodologies with a correct evaluation of results are the indications that emerge from this research, together with the necessity of a strong involvement of students and professors. The aim of GHE is to promote in students professional and life choices coherent with the spirit of GH, which means to promote ethical positioning and social responsibility. GHE allows a reappraisal of the social vision of medicine represented, for example, at the end of the 20th by Rudolf Virchow who remarked: “Medicine is a social science, and politics is nothing but medicine on a grand scale” [40].

## Data Availability

The datasets used and/or analysed during the current study are available from the corresponding author on reasonable request.

## Abbreviations

GH: Global health
GHE: Global health education
INGHE: Italian Network for Global Health Education

## References

[1] Urkin J, Alkan M, Henkin Y, Baram S, Deckelbaum R, Cooper P, Margolis C (2001). Integrating Global Health and medicine into the medical curriculum. Educ Health.

[2] Bateman C, Baker T, Hoornenborg E, Ericsson U (2001). 2001. Bringing global issues to medical teaching. Lancet.

[3] Drain PK, Primack A, Hunt DD, Fawzi WW, Holmes KK, Gardner P (2007). Global health in medical education: a call for more training and opportunities. Acad Med.

[4] Sanson-Fisher RW, Williams N, Outram S (2008). Health inequities: the need for action by schools of medicine. Med Teach.

[5] Battat R, Seidman G, Chadi N, Chanda M, Nehme J, Hulme J, Li A, Faridi N, Brewer T (2010). Global Health competencies and approaches in medical education: a literature review. BMC Med Educ.

[6] Bozorgmehr K, Menzel-Severing J, Schubert K (2010). Global Health education: a cross-sectional study among German medical students to identify needs, deficits and potential benefits (part 2 of 2: knowledge gaps and potential benefits). BMC Med Educ.

[7] Bozorgmehr K, Schubert K, Menzel-Severing J (2010). Global Health education: a cross-sectional study among German medical students to identify needs, deficits and potential benefits (part 1 of 2: mobility patterns & educational needs and demands). BMC Med Educ.

[8] Hanson L (2010). Global citizenship, Global Health, and the internationalization of curriculum: a study of transformative potential. J Stud Int Educ.

[9] Rowson M, Merriel A, Hughes R, Johnson O, Maini A, Martin S, Martineau F, Miranda JJ, Pollit V, Wake R, Willott C, Yudkin J (2012). The Evolution of Global Health Teaching in Undergraduate Medical Curricula. Glob Health.

[10] Rowson M, Willott C, Hughes R, Maini A, Martin S, Miranda JJ, Pollit V, Merriel A, Wake R, Yudkin J (2012). Conceptualising global health: theoretical issues and their relevance for teaching. Glob Health.

[11] Adams L, Wagner C, Nutt C, Binagwaho A (2016). The future of global health education: training for equity in global health. BMC Med Educ.

[12] Kaffes I, Moser F, Pham M, Oetjen A, Fehling M (2016). Global health education in Germany: an analysis of current capacity, needs and barriers. BMC Med Educ.

[13] Sklar D (2016). Global Health education in a changing world: the next new conversations topic. Acad Med.

[14] Woodward-Kron R (2016). Putting Population and Global Health on the Agenda of Health Professionals. J Public Health Res.

[15] Drain PK, Mock C, Toole D (2017). The emergence of undergraduate majors in Global Health: systematic review of programs and recommendations for future directions. Am J Trop Med Hyg.

[16] Litzelman D, Gardner A, Einterz R, Owiti P, Wambui C, Huskins J, Schmitt-Wendholt K, Stone G, Ayuo P, Inui T, Umoren R (2017). On becoming a global citizen: transformative learning through Global Health experiences. Ann Global Health.

[17] Peluso M, van Schalkwyk S, Kellett A, Brewer TF, Clarfield AM, Davies D, Garg B, Greensweig T, Hafler J, Hou J, Maley M, Mayanja-Kizza H, Pemba S, Samaan J, Schoenbaum S, Sethia B, Uribe JP, Margolis CZ, Rohrbaugh RM. Reframing undergraduate medical education in global health: rationale and key principles from the Bellagio Global Health education initiative. Med Teach. 2017. 10.1080/0142159X.2017.1301654. 10.1080/0142159X.2017.1301654 28362131

[18] Sklar DP (2018). Disparities, health inequities, and vulnerable populations: will academic medicine meet the challenge?. Acad Med.

[19] Amerson R (2019). Preparing Undergraduates for the Global Future of Health Care. Ann Global Health.

[20] Kasper J, Greene JA, Farmer PE, Jones DS (2016). All health is Global Health, all medicine is social medicine: integrating the social sciences into the preclinical curriculum. Acad Med.

[21] Blum N, Berlin A, Isaacs A, Burch W, Willott C (2019). Medical students as global citizens: a qualitative study of medical students’ views on global health teaching within the undergraduate medical curriculum. BMC Med Educ.

[22] Peluso MJ, DeLuca MA, Dagna L, Garg B, Hafler JP, Kiguli-Malwadde E, Mayanja-Kizza H, Maley MA, Rohrbaugh RM. Socially Accountable Global Health Education Amidst Political Uncertainty and Reactionary Nationalism: A Value Proposition and Recommendations for Action. Ann Global Health. 2019;85, 118, 1–6(1). 10.5334/aogh.2569. 10.5334/aogh.2569 PMC6729109 31490030

[23] Havemann M, Bösner S (2018). Global Health as “umbrella term” – a qualitative study among Global Health teachers in German medical education. Glob Health.

[24] Frenk J, Chen L, Bhutta ZA, Cohen J, Crisp N, Evans T, Fineberg H, Garcia P, Ke Y, Kelley P (2010). 2010. Health professionals for a new century: transforming education to strengthen health systems in an interdependent world. Lancet.

[25] Missoni E, Missoni E, Tediosi F (2013). Global health education in Italy. Education and Global Health Policy and Management.

[26] Missoni E, Tediosi F, Pacileo G, Gautier L (2014). Italy's contribution to global health: the need for a paradigm shift. Glob Health.

[27] Bruno S, Silvestrini G, Carovillano S, Rinaldi A, Civitelli G, Frisicale E, Marceca M, Tarsitani G, Ricciardi W (2011). e Rete Italiana per l’Insegnamento della Salute Globale (RIISG). L’insegnamento della Salute Globale nelle Facoltà di Medicina e Chirurgia in Italia: l’offerta formativa nel triennio 2007–2010. Ann Ig.

[28] Civitelli G, Tarsitani G, Rinaldi A, Marceca M (2020). Medical education: an Italian contribution to the discussion on global health education. Glob Health.

[29] Wennerstrom A, Gibson J, Krane K (2018). From classroom to community: the impact of a non-clinical clerkship on fourth-year medical students’ ability to address social determinants of health. Med Sci Educ.

[30] Crampton P, Hetherington J, McLachlan J, Illing J (2016). Learning in underserved UK areas: a novel approach. Clin Teach.

[31] Civitelli G, Familiari G, Rinaldi A, Marceca M, Tarsitani G (2015). RIISG. Responsabilità sociale, salute e formazione in medicina. La proposta della RIISG e un’esperienza con i richiedenti protezione internazionale e rifugiati presso la Sapienza Università di Roma. Med Chir.

[32] Asgary R (2016). Graduate public health training in healthcare of refugee asylum seekers and clinical human rights: evaluation of an innovative curriculum. Int J Public Health.

[33] Afkhami A (2016). Can academic medicine Lead the way in the refugee crisis?. Acad Med.

[34] Duke P, Brunger F, Ohle E (2015). Morning in refugee health: an introduction for medical students. Int J Migr Health Soc Care.

[35] Pottie K, Hostland S (2007). Health advocacy for refugees: medical student primer for competence in cultural matters and global health. Can Fam Physician.

[36] Brooker R, Hu W, Reath J (2018). Medical student experiences in prison health services and social cognitive career choice: a qualitative study. BMC Med Educ.

[37] Stys D, Hopman W, Carpenter J (2013). What is the value of global health electives during medical school?. Med Teach.

[38] Boscardin CK, Grbic D, Grumbach K, O’Sullivan P (2014). Educational and individual factors associated with positive change in and reaffirmation of medical students’ intention to practice in underserved areas. Acad Med.

[39] O'Brien M, Garland J, Murphy K, Shuman S, Whitaker R, Larson S (2014). Training medical students in the social determinants of health: the health scholars program at Puentes de Salud. Adv Med Educ Pract.

[40] Virchow, R. ‘Der Armenarzt’, 125–7. In: Collected Essays on Public Health and Epidemiology (CEPHE). 2 volumes. Edited and translated by L J Rather. Canton, Mass.: Watson Publishing International, 1985.

